# Biosemantic graph transfer learning for cross-patient cell type annotation and cell–cell interaction analysis in tumor single-cell RNA sequencing

**DOI:** 10.1093/bib/bbag587

**Published:** 2026-11-13

**Authors:** Yue-Chao Li, Leon Wong, Hai-Ru You, Xin-Fei Wang, Meng-Meng Wei, Lei Wang, Zhi-An Huang, Yu-An Huang, Zhu-Hong You

**Affiliations:** School of Computer Science, Northwestern Polytechnical University, No.1 Dongxiang Road, Chang'an District, Xi'an 710129, Shaanxi, China; College of Big Data and Internet, Shenzhen Technology University, No.3002 Lantian Road, Pingshan District, Shenzhen 518118, Guangdong, China; School of Computer Science, Northwestern Polytechnical University, No.1 Dongxiang Road, Chang'an District, Xi'an 710129, Shaanxi, China; College of Computer Science and Technology, Jilin University, No.2699 Qianjin Street, Chaoyang District, Changchun 130012, Jilin, China; School of Computer Science and Technology, China University of Mining and Technology, No.1 Daxue Road, Tongshan District, Xuzhou 221116, Jiangsu, China; School of Computer Science and Technology, China University of Mining and Technology, No.1 Daxue Road, Tongshan District, Xuzhou 221116, Jiangsu, China; Research Office, City University of Hong Kong (Dongguan), No.8 Gaoxiong Road, Songshan Lake High-tech Industrial Development Zone, Dongguan 523808, Guangdong, China; School of Computer Science, Northwestern Polytechnical University, No.1 Dongxiang Road, Chang'an District, Xi'an 710129, Shaanxi, China; Research and Development Institute, Northwestern Polytechnical University, No.45 Gaoxin South 9th Road, Nanshan District, Shenzhen 518063, Guangdong, China; School of Computer Science, Northwestern Polytechnical University, No.1 Dongxiang Road, Chang'an District, Xi'an 710129, Shaanxi, China

**Keywords:** single-cell RNA sequencing, tumor microenvironment, cell type annotation, cell–cell communication, ligand-receptor interactions

## Abstract

The exploration of cellular heterogeneity within the tumor microenvironment (TME) via single-cell RNA sequencing (scRNA-seq) is essential for understanding cancer progression and response to therapy. Current scRNA-seq approaches, however, lack spatial context and rely on incomplete datasets of ligand–receptor interactions, limiting accurate cell type annotation and cell–cell communication inference. This study addresses these challenges using a novel graph neural network model that enhances cell-type prediction and cell-interaction analysis. Our study utilized a dataset consisting of 49 024 cells from 19 patients across three cancer types: leukemia, breast invasive carcinoma, and colorectal cancer. The proposed scGSL model demonstrated robust performance, achieving an average accuracy of 84.83%, precision of 86.23%, recall of 81.51%, and an F1 score of 80.92% across all datasets. These metrics represent a significant enhancement over existing methods, which typically exhibit lower performance metrics. Additionally, by reviewing existing literature on gene interactions within the TME, the scGSL model proves to robustly identify biologically meaningful gene interactions in an unsupervised manner, validated by significant expression differences in key gene pairs across various cancers. The source code and data used in this study can be found at https://github.com/LiYuechao1998/scGSL.

## Introduction

Single-cell RNA sequencing (scRNA-seq) technology has revolutionized our understanding of cellular heterogeneity within the tumor microenvironment (TME), a complex ecosystem integral to cancer progression and therapeutic response. The TME consists of a diverse array of cellular components including immune cells, endothelial cells, and stromal cells, alongside molecular components such as signaling molecules and extracellular matrix proteins. These elements do not exist in isolation. Instead, they engage in extensive cross talk that significantly influences tumor behavior and patient outcomes [1, 2]. Through scRNA-seq, the intricate cellular interactions and functional heterogeneity that traditional bulk sequencing methods often obscure are now illuminated, providing insights into the unique transcriptional profiles of individual cells [3–5]. This intricate detail enables the identification of specific subpopulations of immune cells that may be uniquely associated with either tumorigenesis or tumor suppression. It also delineates the role of stromal cells like fibroblasts in supporting tumor growth through the secretion of matrix metalloproteinases and other enzymes that facilitate tumor invasion and metastasis. By offering a clearer view of the cellular landscape within the TME, scRNA-seq facilitates a deeper understanding of how these diverse cell types contribute to cancer dynamics and resistance, potentially leading to more effective therapeutic strategies.

However, a significant challenge of scRNA-seq is its inability to retain spatial context of the cells, necessitating subsequent computational analysis for cell type annotation, and the inference of intercellular communication. These analyses are crucial for constructing a comprehensive map of the cellular interactions within the TME, which can reveal potential targets for therapeutic intervention. By understanding how different cell types within the TME communicate and contribute to cancer progression, scRNA-seq enhances the understanding of cancer biology and opens up new avenues for targeted therapy.

While scRNA-seq captures gene expression at the individual cell level, methods for analyzing cell–cell communication (CCC) predominantly focus on interactions between cell types rather than individual cells. These methods fundamentally rely on ligand–receptor interactions (LRIs) to score signaling communication between different cell types. LRIs involve a ligand, typically a protein or small molecule secreted by one cell, binding to a specific receptor on another cell. This binding initiates a cascade of signals within the receiving cell, thereby facilitating communication between cells. LRIs are crucial for explaining how cells influence each other in biological contexts, forming the basis of intercellular communication.

As summarized by Peng *et al*. [6], existing computational approaches for inferring CCC are categorized into three main types: (i) network-based scoring methods, including CCCExplorer [7] and NicheNet [8]; (ii) machine learning prediction techniques, such as PyMINEr [9], SoptSC [10], and SingleCellSignalR [11]; and (iii) spatial information-based inference tools like CellTalker [12], SpaOTsc [13], and histoCAT [14]. The computational pipeline for these methods typically begins with cell type annotation or cell clustering, followed by the organization of original LRI data. Gene expression values of ligands and receptors are used to compute interaction scores for each pair mediating between two cell types, and these scores are aggregated to assess the overall state of communication between them [15]. Each method has its strengths and weaknesses when compared: network-based scoring and machine learning approaches heavily rely on the curation of LRIs and their downstream pathways, whereas spatial information-based inference is limited to spatial datasets and cannot be applied to non-spatial transcriptomics data. CCC inference is increasingly viewed as a data-driven analysis task that varies with different techniques and relies on diverse scoring approaches to determine cell-type specificity, yet the absence of a “gold standard” dataset makes it challenging to evaluate and standardize these methods.

However, existing CCC inference methods universally confront several significant issues. The acquisition of LRIs relies heavily on biological experiments, leading to an incomplete dataset where some interactions are missing, such as ligands like IL17D that lack known receptors and receptors like RELT, NGFR, and TROY from the TNF receptor family that lack known ligands. This incompleteness can introduce biases, particularly affecting rare cell types. Moreover, LRIs are not tumor-specific, implying that in TME scenarios, all known LRIs are considered equally critical across all cell types, potentially leading to an overemphasis on certain interactions. Most CCC inference techniques focus on proteinaceous ligands and receptors, neglecting other important signaling cofactors such as stimulatory and inhibitory membrane-bound co-receptors, along with soluble agonists and antagonists. Beyond the shortcomings related to LRIs, another challenge lies in the dependency of CCC inference methods on cell-type identification, heavily relying on tools like Seurat [16], SingleR [17], Cellassign [18], and scCATCH [19]. However, these methods have an accuracy rate of ~80% and still require significant improvement.

Network-based computational analysis has demonstrated its utility across diverse biomedical problems, including the extraction of drug–disease correlations from incomplete human interactomes, the discovery of cancer-associated DNA methylation patterns for long non-coding RNAs, and the identification of protein complexes in protein interaction networks [20–22]. In single-cell analysis, Wu *et al*. further developed a network-based integrative method that learns cell–cell similarity graphs from transcriptomic and epigenomic profiles for cell-type identification [23]. Building on these advances, recent cell-type identification methods have increasingly constructed cell–cell graphs and employed graph neural networks (GNNs) for node-label prediction. For example, scGCN [24] constructs its graph by calculating similarities between cell expression profiles and applying sparsification. scGNN incorporates interaction probability scores derived from CellChat [25] and LRI to assign weights to graph edges. scPML [26] calculates edge weights using AUCell [27] scores of gene signaling pathways obtained from databases such as Kyoto Encyclopedia of Genes and Genomes (KEGG) [28]. Despite their different graph-construction strategies, these methods consistently demonstrate that modeling informative relationships between cells can improve cell-type prediction.

Cell type annotation and cell–cell relationship modeling are methodologically connected through the graph structure used for cellular information propagation [29, 30]. In graph-based annotation, the adjacency structure determines which neighboring cells contribute to the representation of each cell. However, an initial graph constructed solely from transcriptomic similarity may contain noisy or biologically inconsistent connections because of dropout events, technical variation, and inter-patient heterogeneity. Refining this graph according to cell-type consistency, transcriptional smoothness, sparsity, and low-rank structure can provide a more reliable neighborhood context for GNN-based message propagation, thereby improving the cellular representations used for cross-patient cell-type prediction. Conversely, after cross-patient annotation, the learned representations and predicted cellular identities of query cells provide complementary information for subsequent graph refinement: the learned embeddings encode continuous and transferable transcriptomic characteristics, whereas the predicted labels provide pseudo-supervision for organizing cells with compatible identities.

Based on this sequential and complementary connection, we propose scGSL, a graph-based transfer learning framework for cross-patient cell type annotation and downstream cell–cell relationship analysis in tumor scRNA-seq data. scGSL first applies graph structure learning (GSL) to refine the labeled reference graph, thereby providing a more reliable structural basis for GNN message propagation. Graph domain adaptation (GDA) is subsequently performed between the refined reference graph and the query graph to alleviate inter-patient structural and label-distribution shifts and to predict query-cell types. Finally, the learned query-cell embeddings are used as updated node features, whereas the predicted query-cell types are treated as pseudo-labels to refine the query graph. The resulting graph provides a computational representation of potential relationships among individual cells in the TME.

## Methodology

### Single-cell RNA sequencing datasets

In this study, we utilized a comprehensive scRNA-seq dataset to predict cell types across various diseases. The dataset encompasses a total of 49 024 cells derived from 19 individuals, averaging ~2580 cells per individual. This rich dataset includes cells from patients suffering from three distinct diseases: leukemia, breast invasive carcinoma, and colorectal cancer, which are further subdivided into specific datasets as described below and summarized in Fig. 1, a bar graph illustrating the distribution of cell counts and cell-type proportions among the patients. Specifically, this dataset collection encompasses leukemia samples from GSE142213 (including AYL050 and OX1164) [31], GSE132509 (including PBMMC_1, PBMMC_2, PBMMC_3) [32], and GSE154109 (including P6 and P7); breast invasive carcinoma samples from GSE143423 (including tnbc1 and tnbc6k) [33], E-MTAB-8107 (including sc5rJUQ024, sc5rJUQ026, sc5rJUQ033, sc5rJUQ050, sc5rJUQ060) [34], and GSE150660 (including GSM4555888, GSM4555891) [35]; and colorectal cancer samples from E-MTAB-8107 (including scrEXT009, scrEXT010, scrEXT020).

**Figure 1:**
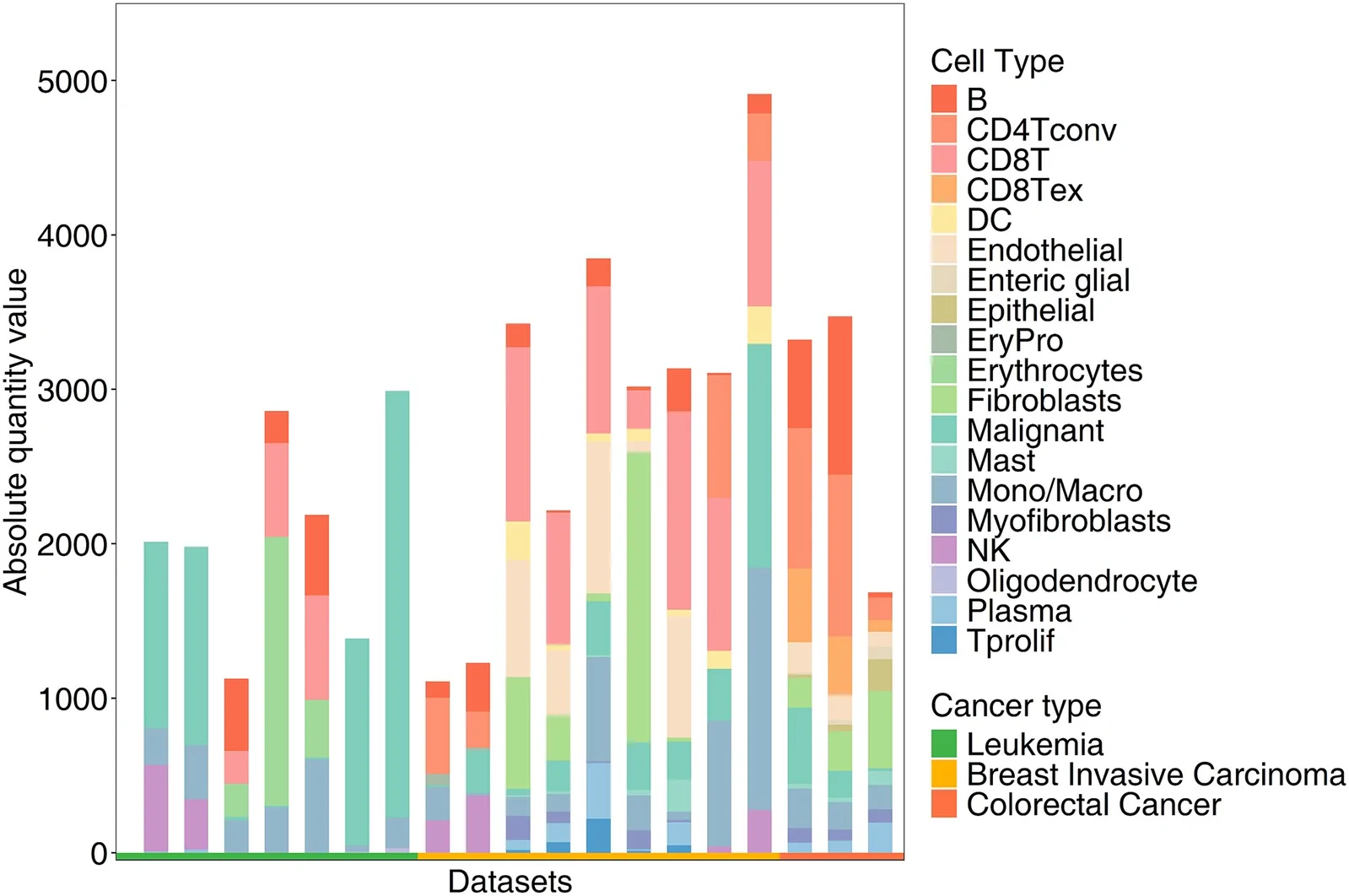
Statistical summary of scRNA-seq dataset.

The cell type annotations provided by the original studies were used as the starting labels. To ensure consistency across patients and cohorts, annotation names were harmonized at the major-cell-type level by standardizing nomenclature and merging synonymous labels that referred to the same biological population. The harmonized labels were used consistently throughout the experiments and are summarized in Fig. 1. Cross-patient transfer tasks were constructed only between patients originating from the same cohort and cancer type; patients from different studies were not directly paired for model training and evaluation. For each directed reference-to-query task, the analysis was restricted to the harmonized cell types shared by the two patients, ensuring that every cell type present in the query dataset was represented in the labeled reference dataset. Therefore, each task followed a pair-specific closed-set annotation setting. The composition of the datasets and the corresponding directed reference-to-query transfer tasks are summarized in Table 1.

**Table 1:** Summary of the scRNA-seq datasets and directed cross-patient transfer tasks used in this study.

Cancer type	Dataset	Number of patients	Number of directed transfer tasks	Cell type count	Number of cells
The leukemia dataset	GSE142213	2	2	4	3994
	GSE132509	3	6	5	6175
	GSE154109	2	2	6	2337
The breast invasive carcinoma dataset	GSE143423	2	2	3	4375
	E-MTAB-8107	5	20	11	15 644
	GSE150660	2	2	7	8020
The colorectal cancer dataset	E-MTAB-8107	3	6	12	8479

Note: For each cohort containing *n* patients, all $n\left(n-1\right)$ ordered reference-to-query transfer directions were evaluated.

### Overview of scGSL model

In this work, we present scGSL, a graph-based transfer learning framework for directed cross-patient cell type annotation and downstream cell–cell relationship analysis using non-spatial tumor scRNA-seq data. Given a labeled reference dataset and an unlabeled query dataset from the same cancer type, scGSL sequentially integrates GSL and GDA [36, 37]. The two modules perform distinct but complementary functions: GSL improves the structural quality of the graph used for cellular message propagation, whereas GDA reduces structural and label-distribution discrepancies between patients to enhance cross-patient transferability.

As illustrated in Fig. 2, scGSL consists of three sequential stages. In the first stage, initial k-nearest neighbor (KNN) graphs are independently constructed for the reference and query datasets. Because reference cell-type labels are available, the reference KNN graph is further refined using the GSL module. This refinement jointly incorporates cell-type consistency, feature smoothness, sparsity, and low-rank constraints, thereby reducing noisy connections and providing a more reliable graph structure for subsequent GNN encoding. In contrast, the query graph remains an initial KNN graph at this stage because query-cell labels are unavailable during annotation.

**Figure 2:**
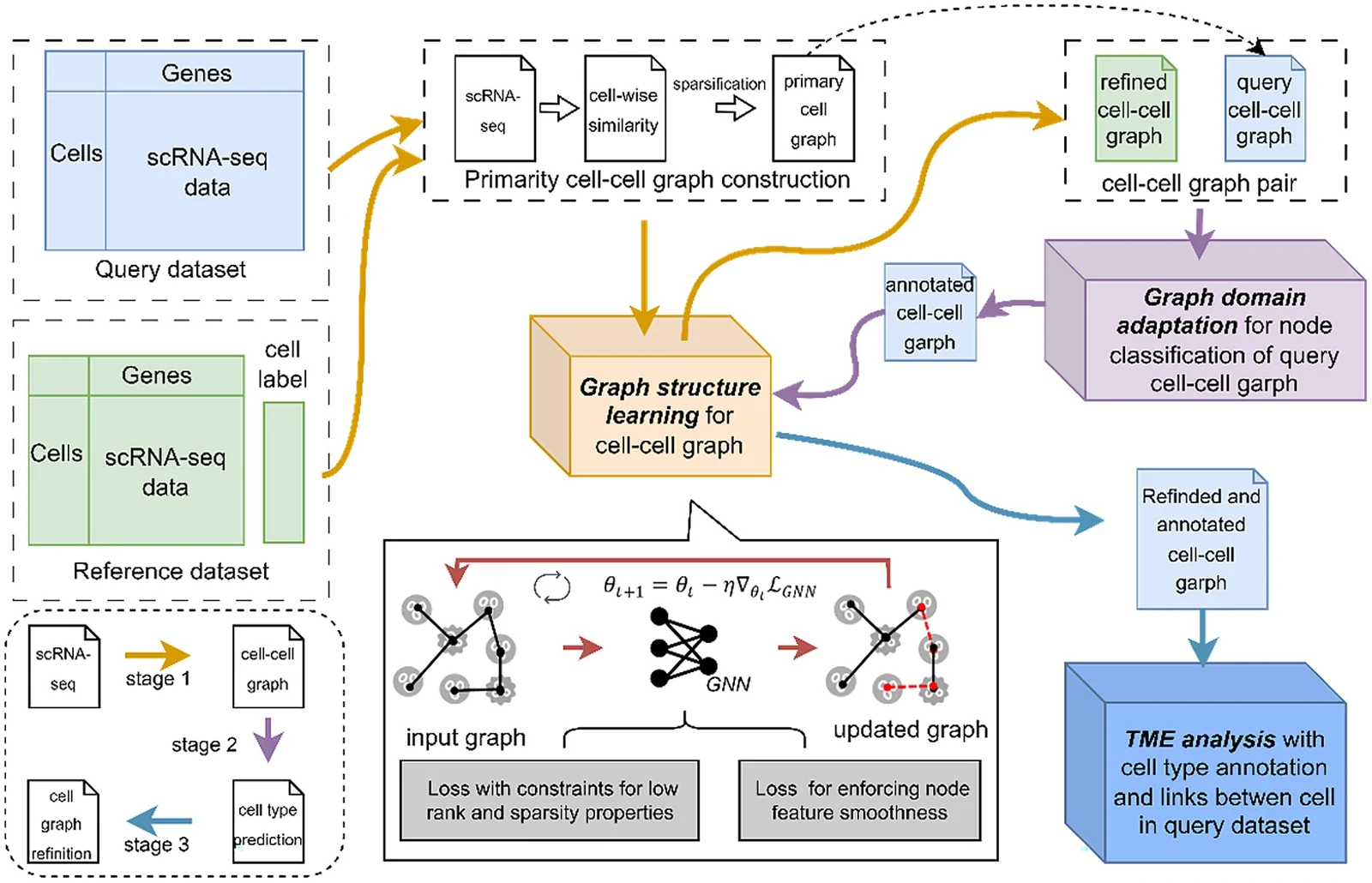
Flowchart of the scGSL model.

In the second stage, the refined reference graph and the initial query graph are provided to the GDA module for cross-patient cell type annotation. The graph convolutional network (GCN) encoder performs graph-based message propagation within the reference and query domains, while the pairwise alignment (PA) framework iteratively adjusts edge-level and class-level importance weights to reduce conditional structural shift (CSS) and label shift (LS) between the two patients. In this manner, the refined reference graph improves the reliability of source-domain cellular representations, whereas GDA enhances the transferability of the learned feature encoder and classifier to the query domain. This stage produces both learned embeddings and predicted cell-type labels for the query cells.

In the third stage, the outputs of cross-patient annotation are used to refine the query graph. Specifically, the learned query-cell embeddings are used as updated node features, whereas the predicted query-cell types are treated as pseudo-labels. The term “predicted labels” refers to the cell-type predictions produced by the classifier in the second stage; these predictions are termed pseudo-labels when they are subsequently used as supervisory information in the final GSL stage. Together with the initial query KNN graph, the updated node features and pseudo-labels are provided to the GSL module to obtain a refined query graph. The resulting graph represents computationally inferred relationships among query cells based on their learned transcriptomic representations, predicted cellular identities, and graph regularization constraints, and is subsequently used for downstream cell–cell relationship analysis. Accordingly, the learned edges should not be interpreted as directly observed physical contacts, spatial proximity, or experimentally confirmed intercellular communication.

### Graph structure learning for cell–cell graphs

Upon acquisition of the scRNA-seq data, quality control was performed by removing cells with fewer than 200 detected genes and genes detected in fewer than three cells. For each reference-query patient pair, the genes shared by the two datasets were first identified, and both expression matrices were restricted to this common gene space. The reference and query matrices were then combined, and the top 3000 highly variable genes were jointly selected from the shared gene space. The pairwise feature-alignment procedure did not introduce any additional library-size normalization or logarithmic transformation. Finally, gene-wise Z-score standardization was jointly applied to the combined reference-query feature matrix, after which the standardized matrix was separated into the corresponding reference and query feature matrices. For each patient dataset, the number of nearest neighbors was set to 0.2% of the total cell count, with at least one neighbor retained. Euclidean distance was used to construct a binary KNN graph without self-connections, and the adjacency matrix was symmetrized by taking the element-wise maximum of the matrix and its transpose, resulting in an undirected graph. Self-connections were subsequently represented by the *A+I* term in the GCN formulation.

In constructing cell–cell interaction graphs from non-spatial transcriptomic scRNA-seq data, challenges such as drop-out events, amplification bias, and insufficient sequencing depth inherently distort the representation of genuine cellular interactions. These issues introduce noise and perturbations that significantly complicate the interpretation of calculated correlations in gene expression profiles, as well as scores derived from signaling pathways or LRIs. We propose that these adverse effects can be mitigated by incorporating cell type information and by preserving two intrinsic characteristics typically observed in real-world graph data: feature smoothness in gene expression profiles and the low-rank and sparsity properties of the cell–cell interaction graph.

Given a cell–cell interaction graph $\mathcal{G}=\left(V,E\right)$, the set *V* consist of *N* nodes, each representing a cell {*v_1_*, *v_2_*,…,*v_N_*} with *E* forming the edges that define cellular interactions captured in the adjacency matrix $A\in{\mathbb{R}}^{N\times N}$. Each node *v_i_* has an associated gene expression profile represented as feature vector *x_i_* in the feature matrix $X\in{\mathbb{R}}^{N\times d}$. Nodes are labeled with their cell type *Y* = {*y_1_*, *y_2_*,…, *y_N_*}, serving as training data for the node classification task. We developed a two-layer GCN, which effectively maps the node embeddings based on cell type information *Y*. This model generates a new graph ${\mathcal{G}}^{\prime }$ with an adjusted adjacency matrix *S* designed to achieve three key objectives: (a) it encapsulates cell type information; (b) it preserves essential graph properties such as low rank and sparsity; and (c) it maintains feature smoothness across neighboring nodes. Specifically, the GCN is structured by the function ${f}_{\theta}\left(X,A\right)=\mathrm{softmax}\left(\hat{A}\sigma \left(\hat{A}X{W}_1\right){W}_2\right)$, where $\hat{A}={\hat{D}}^{-1/2}\left(A+I\right){\hat{D}}^{-1/2}$ and $\hat{D}$ is the diagonal matrix of *A* + *I* with ${\hat{D}}_{ii}=1+{\sum}_j{A}_{ij}$. *σ* represents a *ReLU* activation function. To achieve objective (a), one object function of the GCN can be formulated as:


(1) \begin{equation*} \underset{\theta }{\min }{\mathcal{L}}_{Cls}\left(\theta, A,X,Y\right)=\sum_{v_i\in V}\mathrm{cross}-\mathrm{entropy}\left({f}_{\theta }{\left(X,A\right)}_i,{y}_i\right) \end{equation*}

To enable the GCN to effectively learn the symmetric adjacency matrix *S* (initialized as *A*) of the newly formulated undirected graph ${\mathcal{G}}^{\prime }$ and to achieve objective (b), which entails maintaining essential graph properties of low rank and sparsity, an appropriately designed loss function was incorporated into the model’s training:


(2) \begin{equation*} \underset{S\in{\left[0,1\right]}^{N\times N}}{\arg\ \min\ }{\mathcal{L}}_{graph}={\left\Vert A-S\right\Vert}_F^2+R(S),\kern1.25em \mathrm{s}.\mathrm{t}.,\kern0.5em S={S}^T \end{equation*}

*R*(*S*) represents the constraints applied to *S* to ensure the preservation of properties of low rank and sparsity. According to Candès and Recht [38], it is established that the minimization of the *ℓ*_1_ norm and the nuclear norm of a matrix promotes sparsity and low rank characteristics, respectively. Consequently, Equation (2) can be reformulated as follows:


(3) \begin{equation*} \underset{S\in{\left[0,1\right]}^{N\times N}}{\arg\ \min }{\mathcal{L}}_{graph}={\left\Vert A-S\right\Vert}_F^2+{w}_s{\left\Vert S\right\Vert}_1+{w}_l{\left\Vert S\right\Vert}_{\ast },\mathrm{s}.\mathrm{t}.,\kern0.5em S={S}^T \end{equation*}

where *w_s_* and *w_l_* are predetermined parameters that modulate the influence of sparsity and low rank properties, respectively. In this work, the values of *w_s_* and *w_l_* were respectively set at 5e-4 and 1.5. The third loss function for objective aims to maintain node feature smoothness in the learned graph, which is represented by the following term ${\mathcal{L}}_{node}$


(4) \begin{equation*} {\mathcal{L}}_{node}= tr\left({X}^T LX\right), \end{equation*}

where *L* is the normalized graph Laplacian matrix of *S.* The final objective function is as follows:


(5) \begin{equation*} \arg \underset{S,\theta }{\min}\mathcal{L}={w}_c{\mathcal{L}}_{Cls}+{\mathcal{L}}_{graph}+{w}_n{\mathcal{L}}_{node}, \end{equation*}

*w_c_* and *w*_n_ were set as 0.1 and 1. To solve this loss, we employ an alternating optimization scheme (details provided in Supplementary File S1) to jointly update *θ* and *S.* In the first stage of scGSL, the GSL module is applied to the reference domain using the original gene-expression matrix as node features and the known reference cell types as labels. When GSL is reused in the final query-graph refinement stage, the learned query-cell embeddings are used as the node-feature matrix, the predicted query-cell types are used as pseudo-labels, and the initial query KNN graph is used to initialize the adjacency matrix to be refined. Thus, the two applications of GSL share the same optimization formulation but differ in the node representations and supervisory labels provided to the module.

### Cell type annotation with graph domain adaptation

As an increasing number of GNN algorithms are developed for cell-type prediction using scRNA-seq data, the diversity in the construction of these graphs—whether cell–cell, gene–gene, or cell-gene graphs—and their formulation methods continue to expand. Despite their potential, these methods often face generalization challenges, especially when scRNA-seq data used for inference comes from patients with different health conditions, or when the data is collected using varied methodologies across different studies. This variability can lead to unique interconnection patterns in the data. In the context of GNNs used for single-cell prediction in cell–cell graphs, transfer issues in node classification (i.e. cell type annotation) become apparent, particularly when annotating cell types within the TME of one patient based on a model trained with scRNA-seq data from another. This highlights the inherent biological and contextual differences between individuals. In this work, we introduce for the first time the concept of GDA to address the challenges associated with applying a learned cell–cell graph for cell type annotation across different individuals.

The GSL and GDA modules are connected through the graph structure used by the feature encoder. Specifically, the adjacency matrix refined by GSL is used as the structural input for reference-domain GCN message propagation, whereas the query domain is represented by its initial KNN graph because query-cell labels are unavailable during cross-patient annotation. Based on the representations and predictions obtained from these two graphs, the GDA module estimates edge-level and class-level importance weights to reduce inter-patient CSS and LS. Therefore, GSL primarily improves the reliability of within-domain structural representation, whereas GDA improves the transferability of the learned encoder and classifier across patients. Their sequential integration enables scGSL to benefit from both graph refinement and distribution-shift correction.

For clarity, the subscripts *s* and *t* denote the reference and query domains, respectively. The graphs used for GDA are denoted by ${G}_s=\left({X}_s,{S}_s,{Y}_s\right)$ and ${G}_t=\left({X}_t,{A}_t\right)$, where ${X}_s$ and ${X}_t$ are the node-feature matrices, ${S}_s$ is the GSL-refined source adjacency matrix, ${A}_t$ is the initial target KNN adjacency matrix, and ${Y}_s$ contains the known source-cell labels. In the PA module, *γ* denotes the edge-level weights for CSS correction, *w* and *α* are used to estimate *γ*, and *β* denotes the class-level weights for label-shift correction. The quantities *Σ*, *v*, *C*, and *μ* are used to estimate these edge-level and class-level weighting terms.

Specifically, we train a model to predict cell types from reference scRNA-seq data ${\mathcal{G}}_S=\left({\mathcal{V}}_S,{\mathcal{E}}_S,{\mathcal{X}}_s\right)$ which comprises a feature encoder, $\phi :\chi \to \mathcal{H}$ and a classifier, $g:\mathcal{H}\to Y$. In this work, *ϕ* implemented as a two-layer GCN, similar to the GSL model, and *g* a two-layer Multi-Layer Perceptron. Given the query data ${\mathcal{G}}_T=\left({\mathcal{V}}_T,{\mathcal{E}}_T,{\mathcal{X}}_T\right)$, the objective is to use GDA on this model to minimize the target error ${\varepsilon}_T\left(g\circ \phi \right)$.

In GDA for graphs with the adjacency matrix *A* and node labels *Y*, shifts in graph structure, indicated by ${\mathbb{P}}_S\left(A,Y\right)\ne{\mathbb{P}}_T\left(A,Y\right)$, typically manifest as either CSS, LS, or a combination of both. With decomposition as $\mathbb{P}\left(A,Y\right)=\mathbb{P}\left(A|Y\right)\mathbb{P}(Y)$, CSS and LS are defined as ${\mathbb{P}}_S\left(A|Y\right)\ne{\mathbb{P}}_T\left(A|Y\right)$ and ${\mathbb{P}}_S(Y)\ne{\mathbb{P}}_T(Y)$, respectively. To address these, the GDA framework of PA is employed. Specifically, PA constructs the matrix of density ratio between the target and source graphs $\gamma \in{\mathbb{R}}^{\left|\mathcal{Y}\right|\times \left|\mathcal{Y}\right|}$ to reweight edges for GNN encoding, which can be formulated as follows:


(6) \begin{equation*} {\left[\gamma \right]}_{i,j}=\frac{{\mathbb{P}}_T\left({Y}_v=j|{Y}_u=i,v\in{\mathcal{N}}_u\right)}{{\mathbb{P}}_S\left({Y}_v=j|{Y}_u=i,v\in{\mathcal{N}}_u\right)},\kern0.5em \forall i,j\in \mathcal{Y} \end{equation*}

${\left[\gamma \right]}_{i,j}$ denotes the density ratio from class-*i* nodes to class-*j* nodes between two graphs. To optimize *γ*, PA further decomposes it into another two weights, $w\in{\mathbb{R}}^{\left|\mathcal{Y}\right|\times \left|\mathcal{Y}\right|}$ and $\alpha \in{\mathbb{R}}^{\left|\mathcal{Y}\right|\times 1}$, which are defined as:


(7) \begin{equation*} {\left[w\right]}_{i,j}=\frac{{\mathbb{P}}_T\left({Y}_u=i,{Y}_v=j|{e}_{uv}\in{\mathcal{E}}_T\right)}{{\mathbb{P}}_S\left({Y}_u=i,{Y}_v=j|{e}_{uv}\in{\mathcal{E}}_S\right)}, \end{equation*}

(8) \begin{equation*} {\left[\alpha \right]}_i=\frac{{\mathbb{P}}_T\left({Y}_u=i|{e}_{uv}\in{\mathcal{E}}_T\right)}{{\mathbb{P}}_S\left({Y}_u=i|{e}_{uv}\in{\mathcal{E}}_S\right)},\kern0.5em \forall i,j\in \mathcal{Y}. \end{equation*}

Given *w*, *α* can be derived via:


(9) \begin{equation*} {\left[\alpha \right]}_i=\frac{\sum_{j\in \mathcal{Y}}\left({\left[w\right]}_{i,j}{\mathbb{P}}_S\left({Y}_u=i,{Y}_v=j|{e}_{uv}\in{\mathcal{E}}_S\right)\right)}{{\mathbb{P}}_S\left({Y}_u=i|{e}_{uv}\in{\mathcal{E}}_S\right)}. \end{equation*}

Given w and *α, γ* can then be estimated via:


(10) \begin{equation*} \gamma =\operatorname{diag}{\left(\alpha \right)}^{-1}w. \end{equation*}

To estimate *w*, PA uses pair-wise pseudo-label distribution alignment using the matrix Σ, which represents the joint distribution of predicted and true edge types, and the vector *v,* the distribution of predicted edge types in the target domain. Based on the soft label of node *u* yielded from the classifier *g*, Σ and *v* can be estimated via:


(11) \begin{equation*} {\left[\hat{\Sigma}\right]}_{i,j,{i}^{\prime },{j}^{\prime }}=\frac{1}{\left|{\mathcal{E}}_S\right|}\sum_{e_{uv}\in{\varepsilon}_s,{y}_u={i}^{\prime },{y}_v={j}^{\prime }}{\left[g\left({h}_u^{(L)}\right)\right]}_i\times{\left[g\left({h}_v^{(L)}\right)\right]}_j, \end{equation*}

(12) \begin{equation*} {\left[\hat{v}\right]}_{ij}=\frac{1}{\left|{\mathcal{E}}_T\right|}\sum_{e_{u^{\prime }{v}^{\prime}\in{\varepsilon}_T}}{\left[g\left({h}_{u^{\prime}}^{(L)}\right)\right]}_i\times{\left[g\left({h}_{v^{\prime}}^{(L)}\right)\right]}_j. \end{equation*}

Finally, *w* can be solved via:


(13) \begin{equation*} \underset{w}{\min }{\left\Vert \hat{\Sigma}w-\hat{v}\right\Vert}_2,\kern0.75em \mathrm{s}.\mathrm{t}.\kern0.5em w\ge 0,\mathrm{and}\ \sum_{i,j}{\left[w\right]}_{i,j}{\mathbb{P}}_S\left({Y}_u\!=\!i,{Y}_v\!=\!j|{e}_{uv}\in{\mathcal{E}}_S\right)\!=\!1. \end{equation*}

PA addresses CSS through such an iterative method. It starts by using an estimated *γ* as edge weights in the source graph to reduce the difference between ${\mathbb{P}}_S\left({H}^{(L)}|Y\right)$ and ${\mathbb{P}}_T\left({H}^{(L)}|Y\right)$. With this reduced difference, *w* can be estimated more accurately, leading to better refinement of *γ*. Continuous iterations allow *γ* to progressively improve the conditional alignment, effectively tackling CSS.

To tackle LS, PA calculates the ratio between the source and target label distributions by aligning the node-level pseudo-label distributions, $\beta \in{\mathbb{R}}^{\left|\mathcal{Y}\right|\times 1},$ in which ${\left[\beta \right]}_i=\frac{{\mathbb{P}}_T\left(Y=i\right)}{{\mathbb{P}}_S\left(Y=i\right)},\forall i\in \mathcal{Y}$. Similar to the solution for estimating *w*, *β* is determined by resolving the linear equation *μ* = C*β*, where C represents the confusion matrix from the classifier in the source domain and *μ* denotes the distribution of the predicted labels in the target domain. Based on the soft label of node *u* yielded from the classifier *g*, C, and *μ* are estimated as


(14) \begin{equation*} {\left[\hat{\mathrm{C}}\right]}_{i,{i}^{\prime }}=\frac{1}{\left|{\mathcal{V}}_S\right|}{\sum}_{u\in{\mathcal{V}}_S,{y}_u={i}^{\prime }}{\left[g\left({h}_u^{(L)}\right)\right]}_i \end{equation*}

(15) \begin{equation*} {\left[\hat{\mu}\right]}_i=\frac{1}{\left|{\mathcal{V}}_T\right|}{\sum}_{u\prime \in{\mathcal{V}}_T}{\left[g\left({h}_u^{(L)}\right)\right]}_i \end{equation*}

Ultimately, β can be determined by solving a least squares problem with constraints that ensure a valid target label distribution:


(16) \begin{equation*} \underset{\beta }{\min }{\left\Vert \hat{\mathrm{C}}\beta -\hat{\mu}\right\Vert}_2\kern1em s.t.\kern0.5em \beta \ge 0,\kern0.75em \sum_i{\left[\beta \right]}_i{\mathbb{P}}_S\left(Y=i\right)=1 \end{equation*}

The feature encoder *ϕ* and classifier *g* are trained via the weighted cross-entropy loss:


(17) \begin{equation*} {\mathcal{L}}_C^{\beta }=\frac{1}{\left|{\mathcal{V}}_S\right|}\sum_{v\in{\mathcal{V}}_S}{\left[\beta \right]}_{y_v}\mathrm{cross}-\mathrm{entropy}\left({y}_v,{\hat{y}}_v\right) \end{equation*}

The entire algorithm of PA for addressing the GDA problem in a reference-query pair of cell–cell graphs is shown in Algorithm 1.

**Algorithm 1:** GDA for reference-query cell-cell graphs.

1: **Input** The reference graph ${\mathcal{G}}_S$ with cell type labels *Y_S_*; The query graph ${\mathcal{G}}_T$; The feature encoder *ϕ* and classifier *g*; The numbers of epoch and period for training, *n* and *t*. 2: Initialize *w*, γ and *β*, 3: **while** epoch < *n* or not converged **do** 4: Use *γ* to add weights to reference cell–cell graph 5: Get predicted cell labels of reference graph through ${\hat{Y}}_S=g(\phi ({x}_{S,}{A}_S))$ 6: Update *ϕ* and *g* using Equation (17) 7: **if** epoch ==0(mod *t*) **then** 8: Get predicted cell labels of two graphs, ${\hat{Y}}_S$ and ${\hat{Y}}_T=g\left(\phi \left({x}_{T,}{A}_T\right)\right)$ 9: Update $\hat{\Sigma},\hat{v},\hat{\mathrm{C}}\ \mathrm{and}\ \hat{\mu}$ using Equations (11 and 12) and Equations (14 and 15) 10: Optimize for *w* using Equation (13) and calculate *γ* using Equation (10) 11: Optimize for *β* using Equation (16) 12: **end if** 13: **end while**

## Results

### Predictive performance of the scGSL model across three disease types at the patient level

To evaluate directed cross-patient cell type annotation, we constructed ordered reference-to-query transfer tasks between patients from the same cohort and cancer type. In an experiment denoted as A and B, patient A was used as the labeled reference dataset, whereas patient B was treated as the unlabeled query dataset. For example, PBMMC_1-PBMMC_2 indicates that PBMMC_1 serves as the labeled reference and PBMMC_2 serves as the unlabeled query. The true cell-type labels of the query cells were withheld throughout model training and were used only for performance evaluation. The reverse direction, B and A, was regarded as a separate transfer task. For each directed reference-to-query pair, scGSL was independently initialized and trained, without sharing trained model parameters or query-label information across different tasks. For a cohort containing *n* patients, all $n\left(n-1\right)$ ordered transfer directions were evaluated. Each task was restricted to the harmonized cell types shared by the corresponding reference and query patients and therefore followed a pair-specific closed-set annotation setting. Each directed transfer task was evaluated using four metrics: accuracy (ACC), precision (PRE), recall (REC), and F1 Score (F1). PRE, REC, and F1 were calculated using macro averaging. Figure 3 presents the overall experimental results as violin plots, where each point represents the performance of one independently trained reference-to-query task.

**Figure 3:**
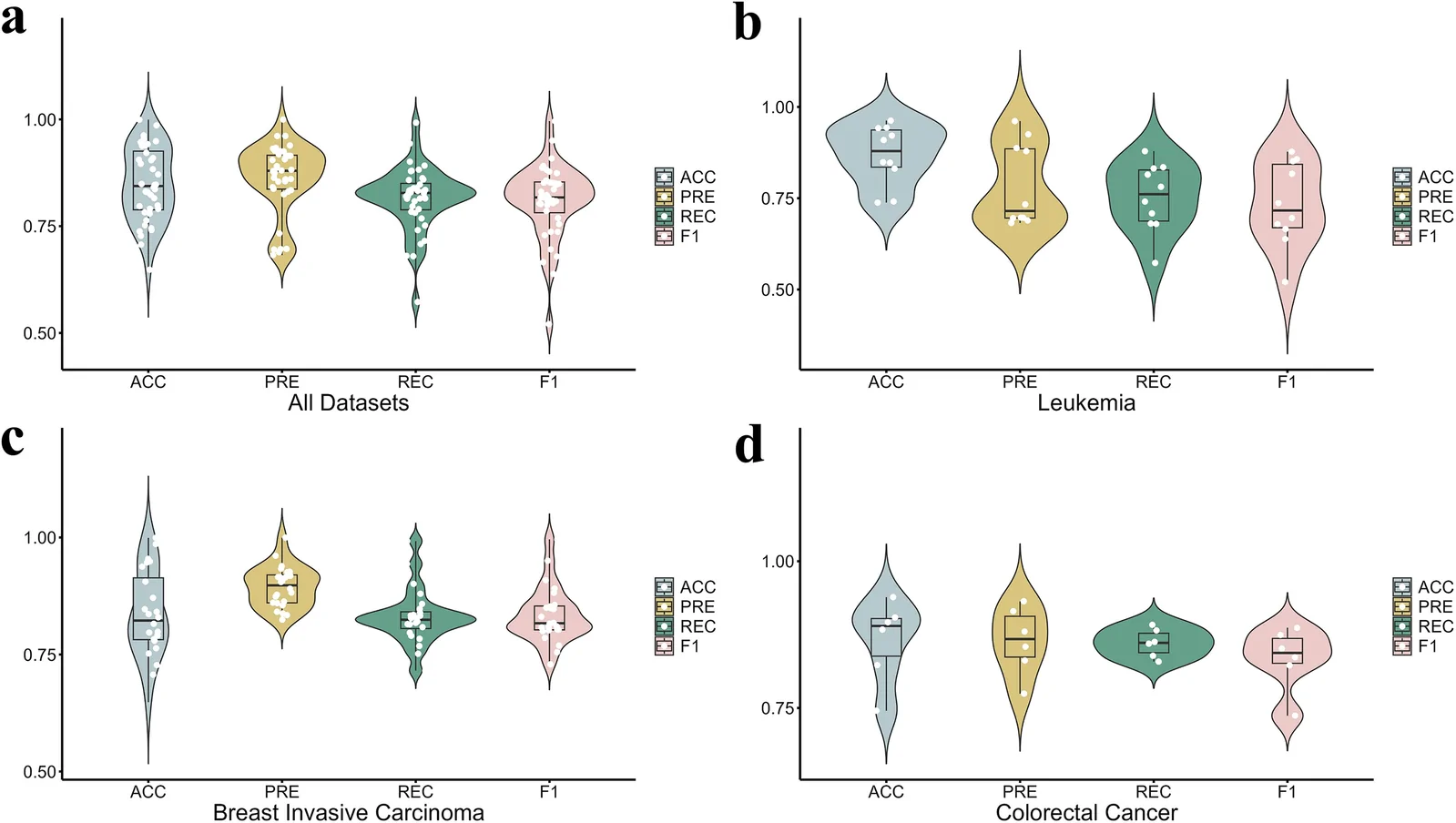
Violin scatter plots of scGSL performance in patient pair experiments.

Overall, the model achieved an average ACC of 84.83%, with PRE at 86.23%, REC at 81.51%, and an F1 of 80.92% across all datasets, indicating robust predictive capabilities. In assessing the scGSL model across three distinctive datasets, each showed varied but generally effective performance metrics. The leukemia dataset showed a robust average ACC of 86.85% and PRE at 78.46%, though it had slightly lower REC and F1 at 75.23% and 73.39%, respectively. The breast invasive carcinoma dataset maintained a balanced performance with an average ACC of 83.56% and a notably high PRE of 89.42%. In the colorectal cancer dataset, the model excelled with an average ACC of 86.52%, complemented by consistent PRE and REC rates of 86.44% and 86.07%. These findings across the datasets underline the scGSL model’s robust predictive capabilities and highlight its potential applicability in diverse clinical scenarios.

### Comparison of scGSL model performance with other models

In the field of scRNA-seq, several computational methods have been developed for cell-type identification, each utilizing different strategies. These include marker-based methods like MarkerCount [39] and CHETAH [40], which rely on known genetic markers; supervised machine learning approaches such as singleCellNet [41], scLearn [42], and scPred [43], which use labeled datasets to train predictive models; reference-based mapping techniques like scmapCell [44], SingleR, and sciBet [45], which match query cells to a reference database; and comprehensive frameworks like Seurat and scClassify [46]. These methods were selected because they represent widely used and complementary cell-type annotation strategies, including marker-based identification, supervised classification, reference-based mapping, and integrative annotation frameworks, thereby enabling a broad comparison with scGSL under the same directed cross-patient evaluation setting. To thoroughly evaluate the efficacy of our scGSL model, we have conducted comparative experiments using these 10 established methods across all available datasets. The results of these comparisons are meticulously presented in Table 2 for the leukemia dataset, Table 3 for the breast invasive carcinoma dataset, and Table 4 for the colorectal cancer dataset. Additionally, a partial comparison of the embeddings learned by these methods is visually displayed in Fig. 4.

**Table 2:** Comparative performance of different models on the leukemia dataset (best performance in bold, second best underlined).

Experiment	Seurat	SingleR	CHETAH	scmapCell	singleCellNet	scLearn	scPred	MarkerCount	scClassify	sciBet	Ours
AYL050-OX1164	**88.49**	81.98	67.59	75.21	81.12	79.00	73.45	65.42	77.89	79.20	83.09
OX1164-AYL050	87.58	87.38	84.45	69.15	84.75	85.25	86.39	76.06	87.43	86.24	**92.15**
PBMMC_1-PBMMC_2	91.71	83.88	80.91	66.85	88.78	92.76	**96.50**	49.93	90.98	75.84	94.20
PBMMC_2-PBMMC_1	88.38	81.99	76.84	67.35	85.09	82.79	**92.46**	79.15	72.58	72.67	90.95
PBMMC_1-PBMMC_3	78.84	83.73	83.27	55.58	77.83	84.32	84.55	83.23	74.31	84.28	**84.87**
PBMMC_3-PBMMC_1	70.72	**91.93**	85.45	61.76	83.67	84.56	73.11	70.81	66.90	84.38	84.74
PBMMC_2-PBMMC_3	94.24	88.25	85.37	69.01	86.79	88.89	75.23	85.19	82.95	84.78	**94.33**
PBMMC_3-PBMMC_2	95.84	96.12	85.38	70.21	95.38	96.19	**97.13**	93.67	95.66	94.27	96.26
P6-P7	62.98	**74.37**	72.01	23.11	71.28	73.47	66.23	67.53	72.09	73.23	73.80
P7-P6	67.60	68.14	**90.79**	19.58	69.04	61.73	69.40	73.19	67.24	67.69	74.10
Average	82.64	83.78	81.21	57.78	82.37	82.90	81.45	74.42	78.80	80.26	**86.85**

**Table 3:** Comparative performance of different models on the breast invasive carcinoma dataset (best performance in bold, second best underlined).

Experiment	Seurat	SingleR	CHETAH	scmapCell	singleCellNet	scLearn	scPred	MarkerCount	scClassify	sciBet	Ours
tnbc1-tnbc6k	98.36	**99.67**	76.31	98.46	94.78	78.62	97.86	75.38	98.03	89.43	98.59
tnbc6k-tnbc1	99.86	99.86	99.64	99.35	**99.93**	**99.93**	99.42	99.49	**99.93**	98.56	**99.93**
sc5rJUQ024-sc5rJUQ026	48.01	67.82	54.20	28.75	71.84	54.83	55.28	49.59	56.32	53.20	**77.93**
sc5rJUQ026-sc5rJUQ024	73.91	81.29	69.09	46.23	75.74	53.97	65.12	48.66	67.66	67.98	**81.55**
sc5rJUQ024-sc5rJUQ033	65.57	**83.78**	54.99	41.68	69.70	69.49	44.15	46.39	65.05	59.67	70.69
sc5rJUQ033-sc5rJUQ024	73.76	77.58	53.39	13.84	73.61	71.37	**79.68**	62.70	72.94	51.90	79.07
sc5rJUQ024-sc5rJUQ050	90.79	93.47	84.13	70.61	90.99	90.49	71.57	86.22	85.55	84.92	**95.43**
sc5rJUQ050-sc5rJUQ024	73.61	76.18	63.05	45.15	76.56	71.48	55.40	67.78	71.28	65.24	**79.68**
sc5rJUQ024-sc5rJUQ060	68.08	79.59	66.36	24.81	78.67	58.39	57.78	45.47	53.03	61.70	**83.64**
sc5rJUQ060-sc5rJUQ024	75.45	80.04	65.21	31.35	78.28	61.88	76.09	62.52	67.83	61.30	**84.68**
sc5rJUQ026-sc5rJUQ033	54.86	47.87	57.90	46.52	63.25	53.04	56.76	41.61	56.91	54.99	**64.81**
sc5rJUQ033-sc5rJUQ026	49.73	80.14	52.35	23.74	73.87	60.47	44.77	63.54	67.96	29.65	**82.94**
sc5rJUQ026-sc5rJUQ050	91.52	94.86	90.56	81.18	92.68	90.62	80.52	84.69	84.00	86.81	**94.90**
sc5rJUQ050-sc5rJUQ026	65.93	**80.91**	57.31	35.15	74.23	70.44	57.18	65.03	65.30	55.32	79.87
sc5rJUQ033-sc5rJUQ050	81.58	85.49	33.83	49.04	90.56	89.56	89.36	89.46	89.70	74.22	**93.77**
sc5rJUQ050-sc5rJUQ033	71.44	55.87	52.81	38.90	63.51	73.99	66.29	38.98	66.27	66.87	**79.03**
sc5rJUQ026-sc5rJUQ060	65.56	**83.04**	71.84	29.88	71.33	53.76	59.25	36.96	67.16	50.32	72.74
sc5rJUQ060-sc5rJUQ026	49.10	73.96	43.32	24.32	69.04	54.15	66.74	60.83	60.97	35.97	**78.25**
sc5rJUQ033-sc5rJUQ060	69.42	79.02	74.49	14.67	78.70	73.47	60.49	57.43	70.60	63.42	**84.12**
sc5rJUQ060-sc5rJUQ033	71.26	71.52	52.34	41.53	74.77	70.92	**79.55**	44.26	72.51	66.50	75.21
sc5rJUQ050-sc5rJUQ060	69.77	76.15	64.48	25.38	74.81	69.90	72.64	60.40	62.21	64.45	**76.34**
sc5rJUQ060-sc5rJUQ050	86.75	93.67	51.33	47.75	85.32	88.44	60.30	84.63	87.87	76.97	**94.67**
GSM4555888-GSM4555891	84.35	**91.96**	72.09	68.04	85.69	72.40	73.97	85.45	62.47	84.82	87.12
GSM4555891-GSM4555888	90.15	82.36	87.13	29.19	89.41	89.31	63.50	83.23	90.41	89.41	**90.57**
Average	73.70	80.67	64.51	43.98	79.05	71.70	68.07	64.20	72.58	66.40	**83.56**

**Table 4:** Comparative performance of different models on the colorectal cancer dataset (best performance in bold, second best underlined).

Experiment	Seurat	SingleR	CHETAH	scmapCell	singleCellNet	scLearn	scPred	MarkerCount	scClassify	sciBet	Ours
scrEXT009-scrEXT010	76.45	85.20	79.12	54.36	89.04	84.13	64.81	76.39	87.10	85.66	**89.61**
scrEXT010-scrEXT009	66.43	83.95	76.93	40.11	79.11	87.50	75.58	84.88	84.97	81.21	**88.35**
scrEXT009-scrEXT020	62.14	90.03	55.85	49.50	73.95	85.46	26.47	69.38	78.16	85.99	**90.45**
scrEXT020-scrEXT009	71.48	77.57	72.69	29.87	70.13	73.26	**77.78**	68.44	70.55	73.02	74.53
scrEXT010-scrEXT020	82.08	92.52	67.77	57.86	82.38	89.97	90.68	75.07	91.04	89.97	**93.89**
scrEXT020-scrEXT010	70.77	64.07	80.94	32.28	80.56	81.23	**86.41**	75.32	80.51	82.21	82.32
Average	71.56	82.22	72.22	44.00	79.19	83.59	70.29	74.92	82.06	83.01	**86.52**

**Figure 4:**
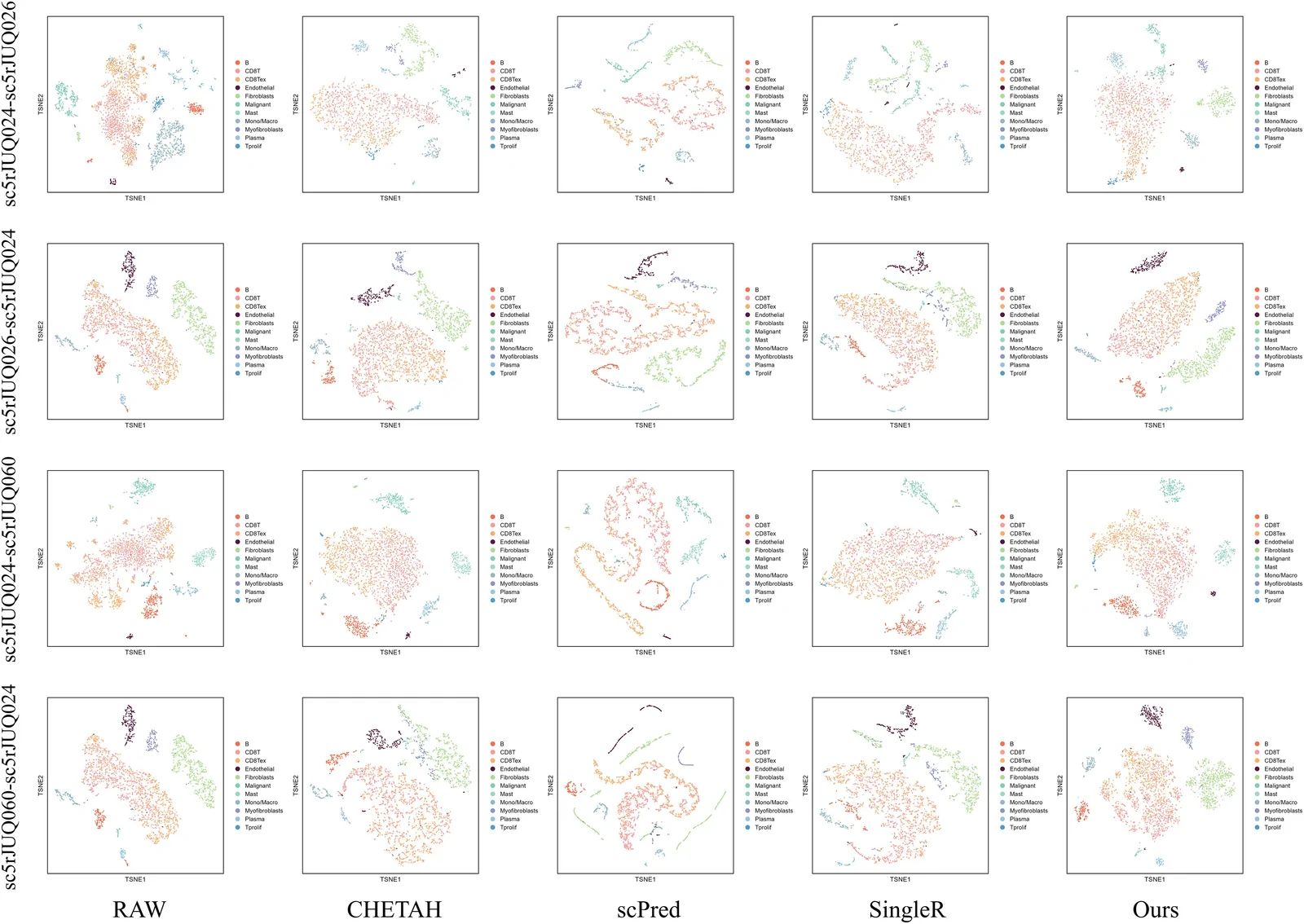
Comparison of cell embeddings and raw data distributions between scGSL and other methods.

In the comparative evaluation across three datasets, our scGSL model consistently demonstrated superior performance. On the leukemia dataset, it achieved the highest average ACC of 86.85%, outperforming the second-best SingleR model by 3.07%. In the breast invasive carcinoma dataset, scGSL led with an average ACC of 83.56%, surpassing SingleR by 2.89%. Similarly, on the colorectal cancer dataset, it recorded the highest average ACC of 86.52%, exceeding the second-best scLearn by 2.93%. These results highlight scGSL’s robust ability to accurately classify cell types, outshining other methods in various cancer contexts. Among the three cancer types, the largest average improvement over the second-best method was observed for leukemia, followed by colorectal cancer and breast invasive carcinoma, with improvements of 3.07, 2.93, and 2.89 percentage points, respectively. Nevertheless, several transfer directions remained relatively challenging, particularly P6-P7, sc5rJUQ026-sc5rJUQ033, and scrEXT020-scrEXT009, which achieved accuracies of 73.80%, 64.81%, and 74.53%, respectively, indicating that annotation difficulty remained dependent on the specific reference-to-query pairing.

### Ablation study for scGSL with different settings

In constructing the cell–cell graph for the scGSL model, HVGs are first selected, followed by the application of the KNN algorithm to establish an initial graph structure. Given that scGSL is a model based on GNN, this section systematically examines the impact of both the selection of the KNN algorithm and the HVG filtering criteria on the predictive performance of scGSL. For the former, Fig. 5a displays violin scatter plots comparing the scGSL model’s performance across different datasets under various KNN settings. The standard setting of K = 0.2% total cell count and embedding size of 128 (K = 0.2%, ES = 128) consistently delivered superior results, achieving the highest average ACC and F1-score. Other tested settings included K = 0.1% and ES = 128, K = 0.5% and ES = 128, K = 0.2% and ES = 64, and K = 0.2% and ES = 256. None of these configurations could match the ACC and F1 results of the standard setting. This emphasizes the effectiveness and robustness of the K = 0.2% and ES = 128 configuration in the scGSL model for optimal cell type classification performance.

**Figure 5:**
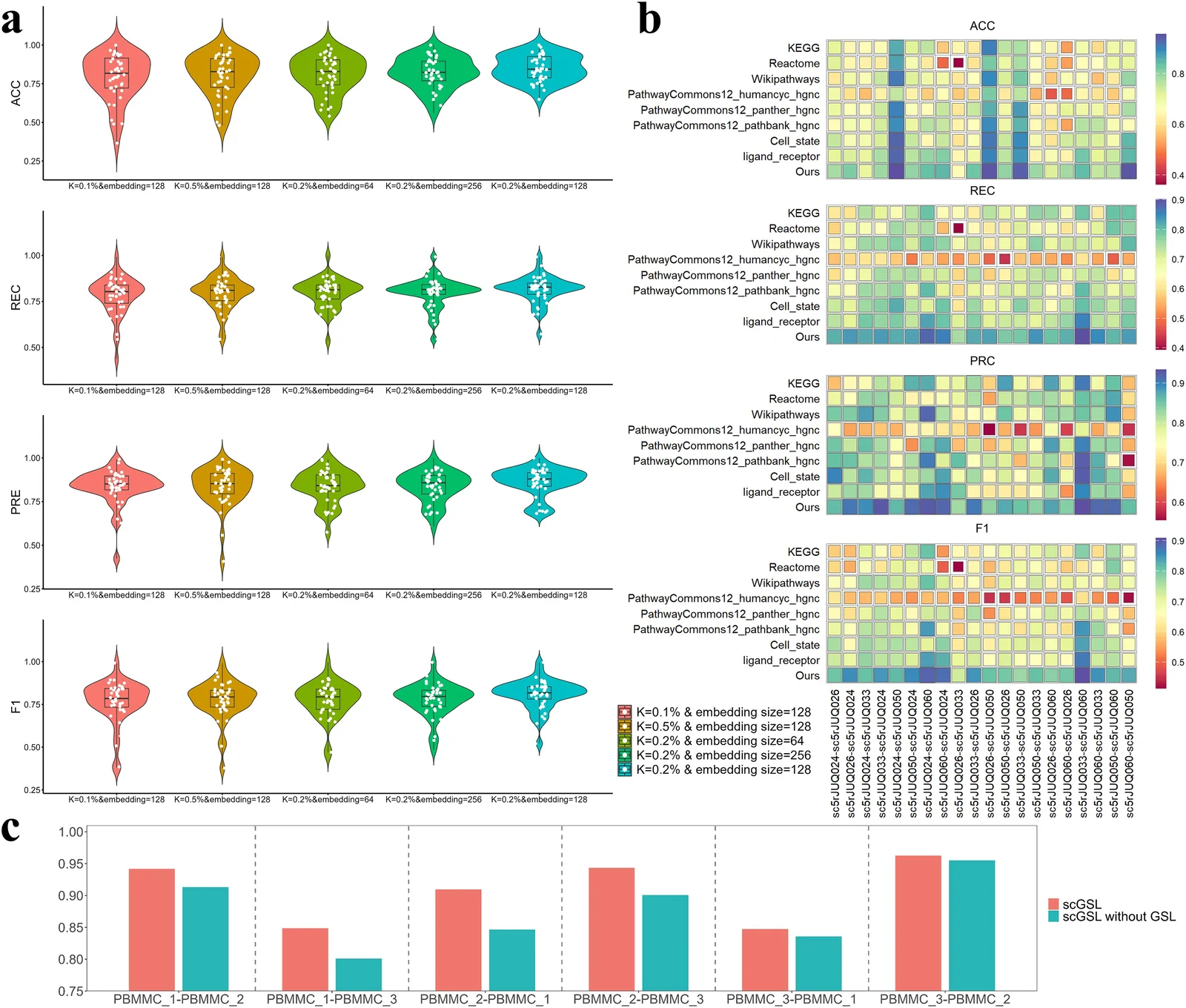
Performance comparison of scGSL under various settings; (a) comparison of performance metrics using varying sparsity levels and neighbor counts during cell–cell graph construction; (b) performance differences when employing various pathway databases for gene filtering; (c) comparative analysis of performance with and without GSL.

To compare different gene selection strategies, we collated gene sets from six gene signaling pathway databases, including KEGG and Reactome, as well as the cell state marker gene database (CancerSEA database) and the LRI database [28, 47–51]. We used the genes found within each of these individual databases as criteria for gene selection, which were then used to construct node features in our graph-based model. Figure 5b showcases a heatmap of the scGSL model’s performance, combining these varied selection approaches on the E-MTAB-8107 dataset, which includes data from 10 unordered patient pairs, corresponding to 20 directed reference-to-query transfer tasks. In our comparative analysis of gene selection strategies across several databases, our method (HVG3000) demonstrated superior performance with an ACC of 81.47%, PRE of 88.39%, REC of 82.49%, and F1 of 82.58%. Figure 5c illustrates a comparison of cell-type prediction accuracies in the GSE132509 dataset, using the scGSL model both with and without the GSL component. The results demonstrate that the inclusion of GSL significantly enhances predictive ACC. Specifically, the average ACC with GSL was 90.89%, compared to 87.55% when GSL was not utilized. This improvement underscores the effectiveness of GSL in enhancing the PRE of cell-type predictions in complex biological datasets. Taken together, these ablation results support the default configuration adopted in scGSL. Among the evaluated settings, the combination of $\mathrm{K}=0.2\%$ and a 128D embedding achieved the best overall performance, HVG3000 outperformed the tested database-specific gene-selection strategies, and incorporating GSL increased the average ACC. Therefore, these settings were selected as the default because they provided the most favorable overall performance across the evaluated tasks.

### Evaluating the interpretability of cell–cell graphs generated from scGSL in the tumor microenvironment

In this section, we explore the interpretability of disease-specific cell–cell graphs derived from scGSL by reviewing existing literature. Specifically, we focus on gene pairs involved in cell–cell interactions within the microenvironments of three diseases: leukemia, breast invasive carcinoma, and colorectal cancer. These gene pairs have been confirmed in prior studies as significant for their roles in intercellular communication within these diseases. Utilizing these validated gene pairs, we assess the statistical significance of these interactions in the constructed cell–cell graphs (visualized in Fig. 6). In the leukemia dataset, the “SPRY1-LAT” and “BSG-ATP2B4” gene pairs within CD8T cells are investigated, confirmed by studies referenced in PMID19915061 [52] and PMID26729804 [53]; in the breast invasive carcinoma dataset, the “ATP2A3-SP1” and “ZAP70-TLN1” gene pairs within CD8T cells are investigated, as established in the literature with PMIDs 22851172 [54] and 20488542 [55]; in the colorectal cancer dataset, the “BIRC5-CASP6” and “PSMC3-SIRT7” gene pairs within malignant cells are investigated, documented in PMIDs 23856250 [56] and 28435470 [57].

**Figure 6:**
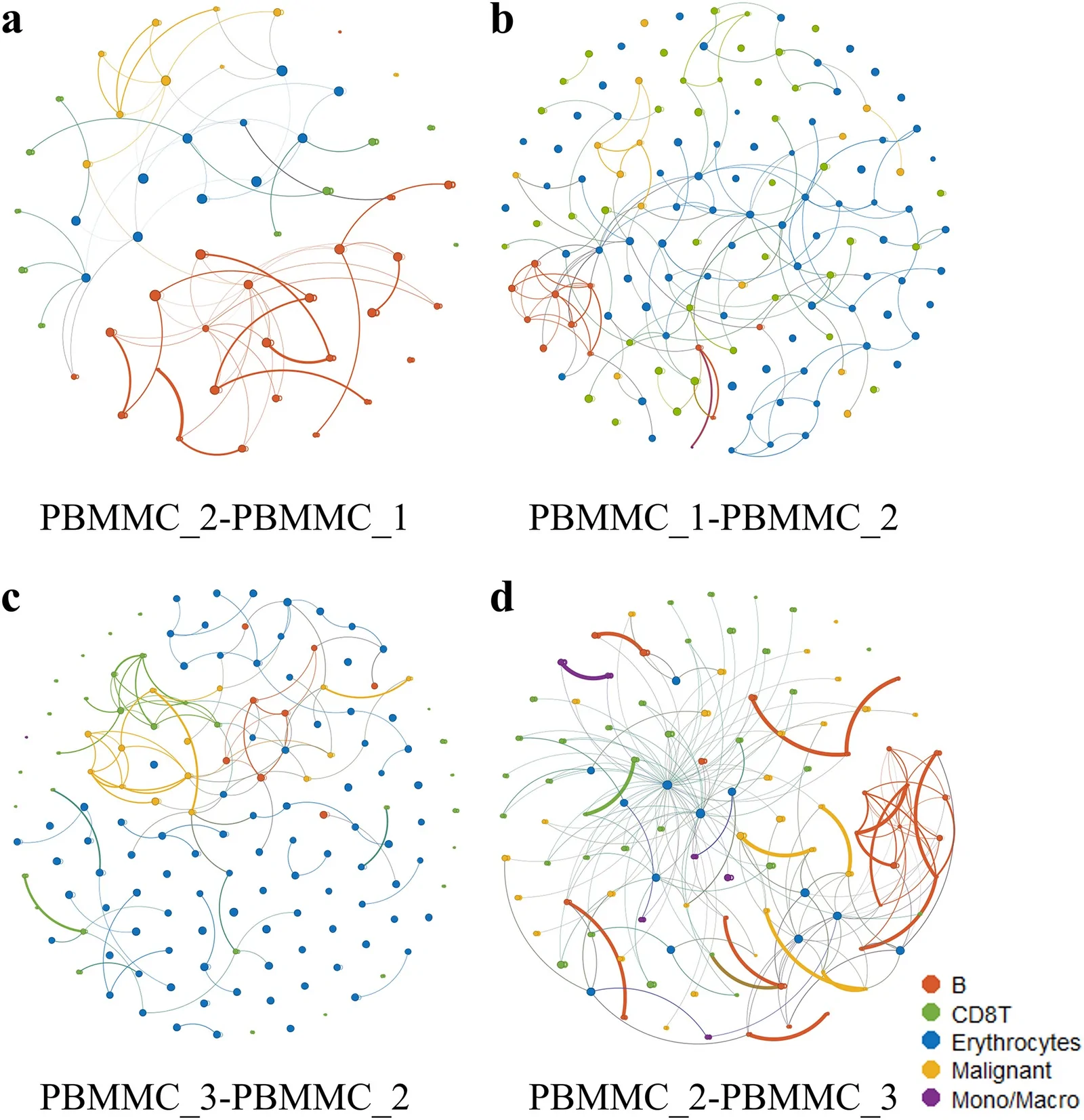
Visualization of cell–cell graphs derived from scGSL on the leukemia dataset.

Specifically, for each query graph obtained from the final GSL stage, we focused on a selected cell-type subgraph, such as the CD8+ T-cell subgraph in the leukemia dataset. Cell pairs within the subgraph were divided into two groups according to the learned graph structure: cell pairs connected by an edge were assigned to the graph-connected group, whereas cell pairs without an edge were assigned to the background group. The Mann–Whitney U test was then performed separately for each individual gene within a literature-supported gene pair to compare its expression distribution between the two groups. Therefore, the *P*-values reported in Tables 5–7 correspond to gene-level expression differences rather than a joint statistical test of the complete gene pair. A significant result suggests that the learned graph captures expression-associated biological patterns involving the corresponding literature-supported genes.

**Table 5:** Statistical significance of known gene interactions for CD8+ T cells in constructed cell–cell graphs on the leukemia dataset (*P*-value <.05 highlighted).

Experiment	SPRY1	LAT	BSG	ATP2B4
PBMMC_1-2	**1.67403e-04**	**9.46e-27**	**7.01e-33**	**7.92883e-04**
PBMMC_2-1	1.15814e-01	3.42939e-01	**3.92324e-03**	**2.50337e-02**
PBMMC_1-3	**1.53503e-03**	**8.07e-05**	**6.01e-05**	**7.62435e-03**
PBMMC_3-1	1.22120e-01	1.46373e-01	**3.58655e-02**	**9.62522e-03**
PBMMC_2-3	**4.06214e-03**	**4.93204e-03**	**1.51e-06**	**1.42773e-02**
PBMMC_3-2	3.44669e-01	**5.62577e-03**	3.48405e-01	1.68556e-01

**Table 6:** Statistical significance of known gene interactions for CD8+ T cells in constructed cell–cell graphs on the breast invasive carcinoma dataset (*P*-value <.05 highlighted).

Experiment	ATP2A3	SP1	ZAP70	TLN1
sc5rJUQ024-26	**1.29373e-04**	**1.27737e-04**	1.17708e-01	**3.21875e-02**
sc5rJUQ026-24	**2.18143e-02**	**6.93e-09**	1.76564e-01	**4.67e-27**
sc5rJUQ024-33	**1.29e-12**	**1.04e-07**	**7.00e-07**	**8.78e-16**
sc5rJUQ033-24	**1.77e-13**	**2.57e-15**	**8.95e-07**	**4.98e-21**

**Table 7:** Statistical significance of known gene interactions for malignant cells in constructed cell–cell graphs on the colorectal cancer dataset (*P*-value <.05 highlighted).

Experiment	BIRC5	CASP6	PSMC3	SIRT7
scrEXT009-10	**2.41032e-02**	**7.15088e-03**	**9.11823e-03**	4.93301e-01
scrEXT010-09	**4.35e-16**	**1.11573e-03**	**1.16e-12**	**1.68677e-02**
scrEXT009-20	**1.19508e-02**	3.99906e-01	3.38843e-01	9.50344e-02
scrEXT020-09	**6.53e-15**	**1.97e-05**	**6.16e-15**	**7.71e-06**

It should be noted that the literature-supported gene pairs evaluated in this analysis were not used during model training. As a result, Tables 5–7 present findings from three different cancer datasets: leukemia, breast invasive carcinoma, and colorectal cancer. In the leukemia dataset, the gene pair “SPRY1-LAT” achieved significant *P*-values (<.05) in 7 out of 12 experiments, while “BSG-ATP2B4” showed significance in 10 out of 12 experiments. In the dataset for breast invasive carcinoma, every experiment involving the gene pair “ATP2A3-SP1” resulted in significant *P*-values, and “ZAP70-TLN1” was significant in 6 out of 8 experiments. In the colorectal cancer dataset, “BIRC5-CASP6” was significant in 7 out of 8 experiments, and “PSMC3-SIRT7” in 5 out of 8. These results underscore the robustness and biological relevance of the unsupervised cell–cell graph approach, as it successfully identified biologically meaningful gene interactions across various types of cancer without prior knowledge of these interactions during the model’s training phase.

## Conclusion

The scGSL model represents a significant advancement in the analysis of non-spatial transcriptomic scRNA-seq data within the context of cancer research. By leveraging GNN and GDA, our model not only enhances the ACC of cell type annotation but also provides a deeper understanding of the intricate cellular interactions within the TME. The ability of the scGSL model to autonomously generate and refine cell–cell interaction graphs based on intrinsic labels ensures a more biologically accurate representation of intercellular communication. This is crucial for identifying potential therapeutic targets and understanding the complex network of interactions that drive tumor progression and resistance to therapies.

Despite its robust performance, the scGSL model faces certain challenges and limitations. One significant challenge is the inherent complexity of TMEs, which can vary widely between different types of cancers and even between patients with the same type of cancer. This variability can affect the generalizability of the model across different datasets and cancer types. Additionally, the reliance on labeled data from specific cancer types for training may limit the model’s applicability to cancers with less available data. Furthermore, the model’s performance is contingent on the quality and completeness of the underlying scRNA-seq data, which can be affected by technical variations and sampling errors. Beyond these general limitations, the biological interpretation of the learned graph should also be treated with caution. Because the datasets used in this study are non-spatial scRNA-seq data, the learned edges represent computationally inferred cell–cell relationships rather than direct physical or spatial interactions. Moreover, the gene-pair analysis assesses gene-level expression differences between graph-connected and background cell pairs and should not be regarded as direct validation of gene–gene interactions or causal intercellular communication.

**Key Points**

- We propose a graph structure learning framework to construct biologically meaningful cell–cell interaction graphs from non-spatial single-cell RNA sequencing data.
- A graph domain adaptation strategy enables robust cross-patient cell type annotation under distribution shifts within the same cancer type.
- The model improves cell-type prediction and cell–cell communication inference by integrating graph learning with biologically informed interaction modeling.
- Extensive experiments demonstrate consistent performance gains, achieving over 84% accuracy, and identifying biologically significant gene interaction patterns.

## Supplementary Material

Supplementary_File_1_bbag587

## Data Availability

The resource, including code, and datasets, are freely available via GitHub at https://github.com/LiYuechao1998/scGSL.

## References

[1] Zhang Y, Li T, Liu Y et al. Supervised pre-training for feature extraction in cell type annotation of single-cell multi-omics data. *Appl Soft Comput* 2025;183:113585. 10.1016/j.asoc.2025.113585

[2] Shi H, Xu T, Li X et al. DRExplainer: quantifiable interpretability in drug response prediction with directed graph convolutional network. *Artif Intell Med* 2025;163:103101. 10.1016/j.artmed.2025.103101 40056540

[3] Yuan H, Liu M, Qiu Y et al. PLNMFG: pseudo-label guided non-negative matrix factorization model with graph constraint for single-cell multi-omics data clustering. *PLoS Comput Biol* 2025;21:e1013375. 10.1371/journal.pcbi.1013375 PMC12416850 40825083

[4] Li Y, Sun Y, Geng A et al. scHilda: hierarchical integration of LLM with KG database for single cell type annotation. *PLoS Comput Biol* 2026;22:e1014291. 10.1371/journal.pcbi.1014291 PMC13175463 42113882

[5] Li T, Wang Z, Liu Y et al. An overview of computational methods in single-cell transcriptomic cell type annotation. *Brief Bioinform* 2025;26:bbaf207. 10.1093/bib/bbaf207 PMC12065632 40347979

[6] Peng L, Wang F, Wang Z et al. Cell–cell communication inference and analysis in the tumour microenvironments from single-cell transcriptomics: data resources and computational strategies. *Brief Bioinform* 2022;23:bbac234. 10.1093/bib/bbac234 35753695

[7] Choi H, Sheng J, Gao D et al. Transcriptome analysis of individual stromal cell populations identifies stroma-tumor crosstalk in mouse lung cancer model. *Cell Rep* 2015;10:1187–201. 10.1016/j.celrep.2015.01.040 25704820

[8] Browaeys R, Saelens W, Saeys Y. NicheNet: modeling intercellular communication by linking ligands to target genes. *Nat Methods* 2020;17:159–62. 10.1038/s41592-019-0667-5 31819264

[9] Tyler SR, Rotti PG, Sun X et al. PyMINEr finds gene and autocrine-paracrine networks from human islet scRNA-seq. *Cell Rep* 2019;26:1951–64.e8. 10.1016/j.celrep.2019.01.063 PMC6394844 30759402

[10] Wang S, Karikomi M, MacLean AL et al. Cell lineage and communication network inference via optimization for single-cell transcriptomics. *Nucleic Acids Res* 2019;47:e66. 10.1093/nar/gkz204 PMC6582411 30923815

[11] Cabello-Aguilar S, Alame M, Kon-Sun-Tack F et al. SingleCellSignalR: inference of intercellular networks from single-cell transcriptomics. *Nucleic Acids Res* 2020;48:e55. 10.1093/nar/gkaa183 PMC7261168 32196115

[12] Cillo AR, Kürten CHL, Tabib T et al. Immune landscape of viral-and carcinogen-driven head and neck cancer. *Immunity* 2020;52:183–99.e9. 10.1016/j.immuni.2019.11.014 PMC7201194 31924475

[13] Cang Z, Nie Q. Inferring spatial and signaling relationships between cells from single cell transcriptomic data. *Nat Commun* 2020;11:2084. 10.1038/s41467-020-15968-5 PMC7190659 32350282

[14] Schapiro D, Jackson HW, Raghuraman S et al. histoCAT: analysis of cell phenotypes and interactions in multiplex image cytometry data. *Nat Methods* 2017;14:873–6. 10.1038/nmeth.4391 PMC5617107 28783155

[15] Cao X, Huang YA, You ZH et al. scPriorGraph: constructing biosemantic cell–cell graphs with prior gene set selection for cell type identification from scRNA-seq data. *Genome Biol* 2024;25:207. 10.1186/s13059-024-03357-w PMC11302842 39103856

[16] Stuart T, Butler A, Hoffman P et al. Comprehensive integration of single-cell data. *Cell* 2019;177:1888–902.e21. 10.1016/j.cell.2019.05.031 PMC6687398 31178118

[17] Aran D, Looney AP, Liu L et al. Reference-based analysis of lung single-cell sequencing reveals a transitional profibrotic macrophage. *Nat Immunol* 2019;20:163–72. 10.1038/s41590-018-0276-y PMC6340744 30643263

[18] Zhang AW, O’Flanagan C, Chavez EA et al. Probabilistic cell-type assignment of single-cell RNA-seq for tumor microenvironment profiling. *Nat Methods* 2019;16:1007–15. 10.1038/s41592-019-0529-1 PMC7485597 31501550

[19] Shao X, Liao J, Lu X et al. scCATCH: automatic annotation on cell types of clusters from single-cell RNA sequencing data. *iScience* 2020;23:100882. 10.1016/j.isci.2020.100882 PMC7031312 32062421

[20] Yu L, Wang B, Ma X et al. The extraction of drug-disease correlations based on module distance in incomplete human interactome. *BMC Syst Biol* 2016;10:111. 10.1186/s12918-016-0364-2 PMC5260043 28155709

[21] Ma X, Yu L, Wang P et al. Discovering DNA methylation patterns for long non-coding RNAs associated with cancer subtypes. *Comput Biol Chem* 2017;69:164–70. 10.1016/j.compbiolchem.2017.03.014 28501295

[22] Ma X, Gao L. Discovering protein complexes in protein interaction networks via exploring the weak ties effect. *BMC Syst Biol* 2012;6:S6. 10.1186/1752-0509-6-S1-S6 PMC3403613 23046740

[23] Wu W, Zhang W, Ma X. Network-based integrative analysis of single-cell transcriptomic and epigenomic data for cell types. *Brief Bioinform* 2022;23:bbab546. 10.1093/bib/bbab546 35043143

[24] Song Q, Su J, Zhang W. scGCN is a graph convolutional networks algorithm for knowledge transfer in single cell omics. *Nat Commun* 2021;12:3826. 10.1038/s41467-021-24172-y PMC8219725 34158507

[25] Jin S, Guerrero-Juarez CF, Zhang L et al. Inference and analysis of cell-cell communication using CellChat. *Nat Commun* 2021;12:1088. 10.1038/s41467-021-21246-9 PMC7889871 33597522

[26] Du Z-H, Du Z-H, Hu W-L et al. scPML: pathway-based multi-view learning for cell type annotation from single-cell RNA-seq data. *Commun Biol* 2023;6:1268. 10.1038/s42003-023-05634-z PMC10721875 38097699

[27] Aibar S, González-Blas CB, Moerman T et al. SCENIC: single-cell regulatory network inference and clustering. *Nat Methods* 2017;14:1083–6. 10.1038/nmeth.4463 PMC5937676 28991892

[28] Kanehisa M, Goto S. KEGG: Kyoto Encyclopedia of Genes and Genomes. *Nucleic Acids Res* 2000;28:27–30. 10.1093/nar/28.1.27 PMC102409 10592173

[29] Wang J, Zou Q, Lin C. A comparison of deep learning-based pre-processing and clustering approaches for single-cell RNA sequencing data. *Brief Bioinform* 2022;23:bbab345. 10.1093/bib/bbab345 34472590

[30] Jiang J, Xu J, Liu Y et al. Dimensionality reduction and visualization of single-cell RNA-seq data with an improved deep variational autoencoder. *Brief Bioinform* 2023;24:bbad152. 10.1093/bib/bbad152 37088976

[31] Di Genua C, Valleta S, Buono M et al. C/EBPα and GATA-2 mutations induce bilineage acute erythroid leukemia through transformation of a neomorphic neutrophil-erythroid progenitor. *Cancer Cell* 2020;37:690–704.e8. 10.1016/j.ccell.2020.03.022 PMC7218711 32330454

[32] Caron M, St-Onge P, Sontag T et al. Single-cell analysis of childhood leukemia reveals a link between developmental states and ribosomal protein expression as a source of intra-individual heterogeneity. *Sci Rep* 2020;10:8079. 10.1038/s41598-020-64929-x PMC7228968 32415257

[33] Bailur JK, McCachren SS, Pendleton K et al. Risk-associated alterations in marrow T cells in pediatric leukemia. *JCI Insight* 2020;5:e140179. 10.1172/jci.insight.140179 PMC7455136 32692727

[34] Qian J, Olbrecht S, Boeckx B et al. A pan-cancer blueprint of the heterogeneous tumor microenvironment revealed by single-cell profiling. *Cell Res* 2020;30:745–62. 10.1038/s41422-020-0355-0 PMC7608385 32561858

[35] Chi Y, Remsik J, Kiseliovas V et al. Cancer cells deploy lipocalin-2 to collect limiting iron in leptomeningeal metastasis. *Science* 2020;369:276–82. 10.1126/science.aaz2193 PMC7816199 32675368

[36] Liu S, Zou D, Zhao H, Li P. Pairwise alignment improves graph domain adaptation. In: Proceedings of the 41st International Conference on Machine Learning (ICML'24). Vienna, Austria: JMLR.org; 2024. Article 1320, 24 pages.

[37] Jin W, Ma Y, Liu X, Tang X, Wang S, Tang J. Graph structure learning for robust graph neural networks. In: Gupta R, Liu Y, Tang J, Prakash BA, editors. Proceedings of the 26th ACM SIGKDD International Conference on Knowledge Discovery & Data Mining (KDD '20). New York, NY, USA: Association for Computing Machinery; 2020. p. 66–74.

[38] Candes E, Recht B. Exact matrix completion via convex optimization. *Commun ACM* 2012;55:111–9. 10.1145/2184319.2184343

[39] Kim H, Lee J, Kang K et al. MarkerCount: a stable, count-based cell type identifier for single-cell RNA-seq experiments. *Comput Struct Biotechnol J* 2022;20:3120–32. 10.1016/j.csbj.2022.06.010 PMC9233224 35782735

[40] De Kanter JK, Lijnzaad P, Candelli T et al. CHETAH: a selective, hierarchical cell type identification method for single-cell RNA sequencing. *Nucleic Acids Res* 2019;47:e95. 10.1093/nar/gkz543 PMC6895264 31226206

[41] Tan Y, Cahan P. SingleCellNet: a computational tool to classify single cell RNA-seq data across platforms and across species. *Cell Syst* 2019;9:207–13. e2. 10.1016/j.cels.2019.06.004 PMC6715530 31377170

[42] Duan B, Zhu C, Chuai G et al. Learning for single-cell assignment. *Sci Adv* 2020;6:eabd0855. 10.1126/sciadv.abd0855 PMC7608777 33127686

[43] Alquicira-Hernandez J, Sathe A, Ji HP et al. scPred: accurate supervised method for cell-type classification from single-cell RNA-seq data. *Genome Biol* 2019;20:1–17. 10.1186/s13059-019-1862-5 PMC6907144 31829268

[44] Kiselev VY, Yiu A, Hemberg M. Scmap: projection of single-cell RNA-seq data across data sets. *Nat Methods* 2018;15:359–62. 10.1038/nmeth.4644 29608555

[45] Li C, Liu B, Kang B et al. SciBet as a portable and fast single cell type identifier. *Nat Commun* 2020;11:1818. 10.1038/s41467-020-15523-2 PMC7156687 32286268

[46] Lin Y, Cao Y, Kim HJ et al. scClassify: sample size estimation and multiscale classification of cells using single and multiple reference. *Mol Syst Biol* 2020;16:e9389. 10.15252/msb.20199389 PMC7306901 32567229

[47] Gillespie M, Jassal B, Stephan R et al. The reactome pathway knowledgebase 2022. *Nucleic Acids Res* 2022;50:D687–92. 10.1093/nar/gkab1028 PMC8689983 34788843

[48] Martens M, Ammar A, Riutta A et al. WikiPathways: connecting communities. *Nucleic Acids Res* 2021;49:D613–21. 10.1093/nar/gkaa1024 PMC7779061 33211851

[49] Cerami EG, Gross BE, Demir E et al. Pathway commons, a web resource for biological pathway data. *Nucleic Acids Res* 2010;39:D685–90. 10.1093/nar/gkq1039 PMC3013659 21071392

[50] Yuan H, Yan M, Zhang G et al. CancerSEA: a cancer single-cell state atlas. *Nucleic Acids Res* 2019;47:D900–8. 10.1093/nar/gky939 PMC6324047 30329142

[51] Yuan Y, Bar-Joseph Z. GCNG: graph convolutional networks for inferring gene interaction from spatial transcriptomics data. *Genome Biol* 2020;21:1–16. 10.1186/s13059-020-02214-w PMC7726911 33303016

[52] Lee JS, Lee JE, Oh YM et al. Recruitment of Sprouty1 to immune synapse regulates T cell receptor signaling. *J Immunol* 2009;183:7178–86. 10.4049/jimmunol.0803799 19915061

[53] Supper V, Schiller HB, Paster W et al. Association of CD147 and calcium exporter PMCA4 uncouples IL-2 expression from early TCR signaling. *J Immunol* 2016;196:1387–99. 10.4049/jimmunol.1501889 26729804

[54] Ghosh S, Adhikary A, Chakraborty S et al. Nifetepimine, a dihydropyrimidone, ensures CD4+ T cell survival in a tumor microenvironment by maneuvering sarco (endo) plasmic reticulum Ca2+ ATPase (SERCA). *J Biol Chem* 2012;287:32881–96. 10.1074/jbc.M112.357889 PMC3463307 22851172

[55] Lin Y-P, Cheng Y-J, Huang J-Y et al. Zap70 controls the interaction of talin with integrin to regulate the chemotactic directionality of T-cell migration. *Mol Immunol* 2010;47:2022–9. 10.1016/j.molimm.2010.04.011 20488542

[56] Végran F, Mary R, Gibeaud A et al. Survivin-3B potentiates immune escape in cancer but also inhibits the toxicity of cancer chemotherapy. *Cancer Res* 2013;73:5391–401. 10.1158/0008-5472.CAN-13-0036 23856250

[57] Tang M, Lu X, Zhang C et al. Downregulation of SIRT7 by 5-fluorouracil induces radiosensitivity in human colorectal cancer. *Theranostics* 2017;7:1346–59. 10.7150/thno.18804 PMC5399598 28435470

