# Supplementary material for: Biosemantic graph transfer learning for cross-patient cell type annotation and cell–cell interaction analysis in tumor single-cell RNA sequencing
Source: Brief Bioinform. 2026 Nov 13;27(6):bbag587. doi: 10.1093/bib/bbag587 (PMC13760016; doi:10.1093/bib/bbag587)
Supplement: Supplementary file 1 [file supplementary_file_1_bbag587.docx]

Biosemantic Graph Transfer Learning for Cross-Patient Cell Type Annotation and Cell-Cell Interaction Analysis in Tumor scRNA-seq

Yue-Chao Li^1^, Leon Wong^2, *^, Hai-Ru You^1^, Xin-Fei Wang^4^, Meng-Meng Wei^5^, Lei Wang^5^, Zhi-An Huang^6^, Yu-An Huang^1,3, *^, Zhu-Hong You^1, *^

^1^ School of Computer Science, Northwestern Polytechnical University, Shaanxi 710129, China

^2^ College of Big Data and Internet, Shenzhen Technology University, Shenzhen, China

^3^ Research & Development Institute of Northwestern Polytechnical University in Shenzhen, Shenzhen, 518063, China

^4^ College of Computer Science and Technology, Jilin University, Changchun, 130012, China

^5^ School of Computer Science and Technology, China University of Mining and Technology, Xuzhou 221116, China

^6^ Research Office, City University of Hong Kong (Dongguan), Dongguan, 523000, China

*corresponding authors

**Updating *θ*.** To update *θ*, we fix *S* and eliminate terms that do not pertain to *θ*. Consequently, the objective function in Equation (5) simplifies to $\mathcal{L}_{Cls}\left( \theta,A,X,Y \right)$, which we can simply learn *θ* via stochastic gradient descent.

**Updating *S*.** To update *S*, we fix *θ* and eliminate terms that do not pertain to *S*. Consequently, the objective function in Equation (5) simplifies to:

$\min_{S} \mathcal{L}\left( S,A \right)+\alpha\left\| S \right\|_{1}+\beta\left\| S \right\|_{*}$ *s.t.*, $S=S^{T}$	(1)

where $\mathcal{L}\left( S,A \right)= \left\| A-S \right\|_{F}^{2}+\gamma\mathcal{L}_{Cls}+\lambda\mathcal{L}_{graph}$ (2)

It should be noted that both the ℓ1 norm and the nuclear norm are inherently non-differentiable. In scenarios where the optimization problem incorporates a singular non-differentiable regularizer *R*(*S*), the application of Forward-Backward splitting methods [1] is advisable. This approach alternates between a gradient descent step and a proximal step, as described below:

$S^{(k)}=\text{prox}_{\eta R}\left( S^{\left( k-1 \right)}-\eta\nabla_{S}\mathcal{L}\left( S,A \right) \right),$	(2)

where 𝜂 represents the learning rate, and prox*R* denotes the proximal operator, defined as follow:

$\text{prox}_{R}\left( Z \right)=\arg\min\frac{1}{2}\left\| S-Z \right\|_{F}^{2}+R(S)$	(3)

Specifically, the proximal operators for the ℓ1 norm and the nuclear norm can be expressed as:

$\text{prox}_{\alpha\left\| \cdot\right\|_{1}}\left( Z \right)=sgn(Z)⨀{(\left| Z \right|-\alpha)}_{+}$ , (4)

$\text{prox}_{\beta\left\| \cdot\right\|_{*}}\left( Z \right)=Udiag{({(\sigma_{i}-\beta)}_{+})}_{i}V^{T}$ , (5)

where *Z* = U*diag*(*σ*_1_,…, *σ*_n_) *V*^T^ is the singular value decomposition of *Z*. To manage the optimization of an objective function that includes two non-differentiable regularizers, Richard et al. [2] propose the Incremental Proximal Descent method, utilizing the aforementioned proximal operators. This method involves cyclically iterating the update process, allowing for the updating of 𝑆 as described below:

$\left\{ \begin{aligned} S^{(k)}=S^{(k-1)}-\eta\cdot\nabla_{S}\left( \mathcal{L}\left( S,A \right) \right), \\ S^{(k)}=\text{prox}_{\eta\beta\left\| \cdot\right\|_{*}}\left( S^{\left( k \right)} \right), \\ S^{(k)}=\text{prox}_{\eta\alpha\left\| \cdot\right\|_{1}}\left( S^{\left( k \right)} \right), \end{aligned} \right.$	(6)

Once we have learned a relaxed version of 𝑆, we project it to satisfy specific constraints. For the symmetry constraint, we define 𝑆 as 𝑆=(𝑆+𝑆^T^)/2. Regarding the constraint that each element 𝑆_𝑖𝑗_ must lie within the range [0, 1], we project any 𝑆_𝑖𝑗_ values less than 0 to 0 and any greater than 1 to 1. We denote these projection procedures as 𝑃_𝑆_(𝑆).

**Training Algorithm:**

***Initialize*** *S*←*A*

***Randomize*** *θ*

***while*** the error has not converged ***do***

$S\leftarrow S-\eta\cdot\nabla_{S}\left( \left\| A-S \right\|_{F}^{2}+{\gamma\mathcal{L}}_{Cls}+\lambda\mathcal{L}_{graph} \right)$

$S\leftarrow\text{prox}_{\eta\beta\left\| \cdot\right\|_{*}}\left( S \right)$

$S\leftarrow\text{prox}_{\eta\alpha\left\| \cdot\right\|_{1}}\left( S \right)$

$S\leftarrow P_{S}\left( S \right)$

***for*** *i* = 1 to τ ***do***

$g\leftarrow\frac{\partial\mathcal{L}_{Cls}(\theta,A,X,Y)}{\partial\theta}$

$\theta\leftarrow\theta-\eta'g$

***Return*** *S*, *θ*

**Reference**

[1] Combettes, Patrick L., and Jean-Christophe Pesquet. "Proximal splitting methods in signal processing." *Fixed-point algorithms for inverse problems in science and engineering* (2011): 185-212.

[2] Raguet, Hugo, Jalal Fadili, and Gabriel Peyré. "A generalized forward-backward splitting." SIAM Journal on Imaging Sciences 6.3 (2013): 1199-1226.
